# Early human trophoblast development: from morphology to function

**DOI:** 10.1007/s00018-022-04377-0

**Published:** 2022-06-05

**Authors:** Martin Gauster, Gerit Moser, Stefan Wernitznig, Nadja Kupper, Berthold Huppertz

**Affiliations:** Division of Cell Biology, Histology and Embryology, Gottfried Schatz Research Center, Medical University of Graz, Neue Stiftingtalstraße 6, 8010 Graz, Austria

**Keywords:** Blastocyst, Trophectoderm, Extravillous trophoblast, Villous trophoblast, Syncytialization

## Abstract

Human pregnancy depends on the proper development of the embryo prior to implantation and the implantation of the embryo into the uterine wall. During the pre-implantation phase, formation of the morula is followed by internalization of blastomeres that differentiate into the pluripotent inner cell mass lineage, while the cells on the surface undergo polarization and differentiate into the trophectoderm of the blastocyst. The trophectoderm mediates apposition and adhesion of the blastocyst to the uterine epithelium. These processes lead to a stable contact between embryonic and maternal tissues, resulting in the formation of a new organ, the placenta. During implantation, the trophectoderm cells start to differentiate and form the basis for multiple specialized trophoblast subpopulations, all of which fulfilling specific key functions in placentation. They either differentiate into polar cells serving typical epithelial functions, or into apolar invasive cells that adapt the uterine wall to progressing pregnancy. The composition of these trophoblast subpopulations is crucial for human placenta development and alterations are suggested to result in placenta-associated pregnancy pathologies. This review article focuses on what is known about very early processes in human reproduction and emphasizes on morphological and functional aspects of early trophoblast differentiation and subpopulations.

## Introduction

Successful human pregnancy strongly depends on crucial mechanisms, such as fertilization of an oocyte, pre-implantation development of the embryo and its implantation into the uterine wall. Embryo implantation is followed by development of the placenta, a tissue of limited life span that fulfills a remarkable array of functions at the maternal–fetal interface to ensure adequate fetal growth. Especially in the early stages of pregnancy, it substitutes fetal organs until they reach full maturity. One of the key functions of the placenta is the transfer of almost all nutrients and gases between mother and fetus [48]. Moreover, the placenta acts as an endocrine organ, secreting a plethora of steroid and protein hormones, metabolic proteins, growth factors, and cytokines to adapt maternal physiology to pregnancy [174]. The key placental cell type involved in all these processes is the trophoblast, a term derived from the two ancient Greek words *trephein* and *blastos*, which mean ‘to feed’ and ‘germinator’.

The trophoblast originates from the trophectoderm (see below) and differentiates into multiple specialized trophoblast subpopulations and phenotypes. They either differentiate into polar cells serving typical epithelial functions, or into apolar invasive cells that remodel and adapt the maternal uterine wall to progressing pregnancy. Differentiation into trophoblast subpopulations and acquisition of diverse phenotypes include molecular changes, such as remodeling of the cytoskeletal composition and a switch of the adhesion molecule pattern, depending on spatiotemporal requirements of the respective cells [70, 111, 127]. Impaired trophoblast differentiation and altered composition of these cell populations in the placenta are associated with placental dysfunction and pregnancy complications that are suggested to give rise to aberrant fetal development and increased risk of long-term development of chronic diseases in the offspring later in life, in a concept known as the Fetal Origins Hypothesis or Developmental Origin of Health and Diseases (DOHaD) [18]. This review article focuses on what is known about very early processes in human reproduction and emphasizes on morphological and functional aspects of early trophoblast subpopulations.

## Trophoblast lineage morphogenesis in the blastocyst

**Fig. 1:**
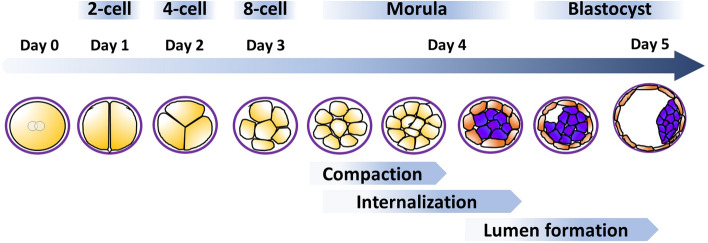
Blastocyst formation. Fertilization of the oocyte gives rise to a diploid zygote (day 0) that undergoes three rounds of cleavage divisions until day 3 post fertilization. Thereafter, compaction of the arising blastomeres starts to arrange cells in a compact cell cluster. By day 4 post fertilization, the morula stage becomes finalized when the embryo is compacted and contains internalized cells (purple), giving rise to the inner cell mass. The outer cells (orange) become the trophectoderm and fluidic uptake leads to a growing lumen (blastocoel), flattening of the outer cell layer and arrangement of the inner cell mass on one pole (embryonic pole). Note that the blastocyst is still surrounded by the zona pellucida, a glycoprotein-rich membrane formed already during oogenesis. Adapted from [60]

In the human, fertilization of the oocyte by a sperm forms a diploid zygote and is the earliest developmental stage. The zygote does not possess any implantation competencies and hence undergoes three rounds of cleavage divisions within days 2–3 post fertilization, which, in contrast to conventional cell divisions, are not accompanied by a significant overall cell growth during the interphase. Hence, the early embryo does not increase in total volume during this stage of development. The cells arising from cleavage divisions are referred to as blastomeres and start to arrange in a compact cluster of cells called morula (Fig. 1).

The early cleavage rounds are frequently prone to errors, including inaccurate cytokinesis or chromosome segregation anomalies. Cytokinesis can be asymmetric and may produce blastomeres of unequal size [164], while chromosome segregation anomalies can partly result from failure to cluster the parental genomes prior to chromosome segregation [31, 35, 154]. Another cytokinesis defect, referred to as direct cleavage, occurs when tripolar mitotic spindles produce three daughter cells instead of two.

In the human, the cleavage stage is followed by the process of compaction, which has recently been suggested as an important checkpoint involved in mechanisms of self-correction [117]. Compaction classically occurs between the 8- and 10-cell stage at 3–4 days post fertilization [89], which is a period of largely underestimated significance. During normal compaction, blastomeres show early signs of polarization, increase the contact area between cells and reduce the surface exposed to the outside to form the compacted morula [60, 140].

A recent study with supernumerary IVF human embryos at day 4 has determined the sequence of developmental events of human embryo polarization in relation to compaction [199]. Accordingly, compacted blastomeres display an apical enrichment of cortical F-actin at the cell-contact free surface, along with a slightly time-delayed polarization of the PAR complex components PARD6 and atypical Protein Kinase C (aPKC). The major regulator of this cell polarity in the human embryo is phospholipase C signaling, specifically via the enzymes PLCB1 and PLCE1, which however, are not directly involved in compaction. Observations in human embryos as well as a 3D human expanded pluripotent stem cell model suggest that acquisition of the apico-basal polarity reinforces the expression of the trophectoderm specific transcription factor GATA3 [172]. While F-actin polarization is not involved in compaction, alternative mechanisms, such as the contractile actomyosin machinery and the calcium-dependent cell–cell adhesion molecule E-cadherin (*CDH1*), have been suggested to move blastomeres closer together forming a tighter structure [3, 173].

Similar to the early stage of cleavage divisions, also during compaction, the embryo is prone to errors, including either complete failure of compaction or less severe defects leading to unequal or partial compaction. Observations from intracytoplasmic sperm injection (ICSI) procedures suggest that unequal compaction, i.e., exclusion of one or more blastomeres from the compacted morula, is associated with reduced developmental competence [51]. Excluded cells are suggested to subsequently contribute to the outer cell mass of the emerging blastocyst or may be excluded entirely from the compacted morula [60]. In this context, unequal compaction has recently been suggested as a potential aneuploidy rescue mechanism, ensuring that defective, aneuploid cells are excluded from mosaic embryos [117]. When excluded, aneuploid blastomeres contribute to the outer cell mass, and they eventually end up in the placenta, which indeed shows a high rate of aneuploidy. Indeed, the placenta has long been considered as a deposition site for eliminated defective cells from embryonic tissues [39].

Beside the important step of compaction, transformation from the morula to the blastocyst stage comprises additional fundamental cellular rearrangements including internalization and lumen formation. During internalization at the time of the fifth cleavage, a subset of blastomeres becomes entirely surrounded by neighboring cells and thus is isolated from the surface. Observations in mouse embryos suggest that cell division may be oriented by alignment of the mitotic spindle vertically to the embryonic surface. Following this, contractility-mediated cell sorting leads to internalization of cells destined to be part of the inner cell mass [60, 113, 124, 191].

This cell positioning is considered a prerequisite for the first lineage formation, guiding the differentiation of internalized cells into the pluripotent inner cell mass lineage that gives rise to all embryonic cells and tissues. Those cells remaining on the surface undergo apico-basal polarization and differentiate into the trophectoderm that is the precursor of a specific subset of cells in the extra-embryonic tissues after implantation, the trophoblast residing in the placenta and the fetal membranes. The polarization process and tight sealing of the trophectoderm enable formation of the blastocoel, a fluid-filled cavity that is generated when fluid is pumped through the water transporter aquaporin 3 (AQP3) of the polarized surface of the trophectoderm into intercellular spaces between trophectoderm cells and cells of the inner cell mass (a process also referred to as cavitation). This way, initial accumulation of fluid leads to swelling of the embryo to almost 10 times its original volume [116], and the increasing pressure eventually breaks down cell–cell contacts and pushes the inner cell mass into one quadrant of the arising blastocyst at 5 days post fertilization [49]. The region where the inner cell mass is attached to the trophectoderm is called the embryonic pole, which is the crucial site for interaction of the blastocyst with the endometrium.

The early blastocyst is still surrounded by the *zona pellucida*, a glycoprotein-rich extracellular matrix already formed during oogenesis. Further uptake of fluid into the blastocoel and an increase in internal hydrostatic pressure lead to flattening of the trophoblast epithelium and a two- to three-fold enlargement of the blastocyst volume [161]. Then, the blastocyst starts to hatch from the *zona pellucida* with the aid of so-called zona-breaker cells, which are described as specialized, plump trophectoderm cells at the point of hatching. Paradoxically, this area usually comprises the abembryonic pole of the blastocyst, where finger-like trophectodermal projections penetrate the *zona pellucida*, and provide hatching-associated factors, such as growth factors, cytokines and zona lysing proteases (e.g., cathepsin), to induce its focal lysis [161, 165]. The blastocyst gradually egresses from the otherwise intact *zona pellucida* and is now prepared for implantation.

## Blastocyst apposition and adhesion—the role of the trophectoderm

Blastocyst formation is synchronized with differentiation of the uterine endometrium under the control of ovarian steroid hormones to reach an appropriate level of maturity. The period when the endometrium shows the highest receptivity for embryo implantation is defined as the implantation window, which occurs in the mid-secretory phase of the menstrual cycle [16, 78]. Due to ethical considerations, our knowledge about initial events of the attachment phase in which the trophectoderm interacts with the lining epithelium of the uterus is very limited and is mostly based on observations from animal models.

However, several novel stem cell-derived 3D models have been established and represent promising tools to study unique stages of human embryo development [172, 197]. A unique model of the human blastocyst that specifically generates cellular analogs of the blastocyst stage with similar developmental sequence has recently been described by Kagawa et al. [95], who efficiently generated blastoids by triple inhibition of the Hippo-, TGF-β- and ERK pathways in cultured naive human pluripotent stem cell aggregates. Inhibition of the Hippo pathway is required to initiate the specification into the trophectoderm, and frees the transcriptional regulator YAP1 to enter the nucleus, which together with transcription factor TEAD is important for the cavitation process. Moreover, this model seems capable to study initial processes involved in human blastocyst attachment, since blastoids are able to attach to and repel cells of a 2D open-faced endometrial layer, mimicking the mid-secretory phase endometrium. Interestingly, this blastoid model suggests that the epiblast, which is embryonic and one of the three lineages generated in this model, induces polar trophectoderm maturation and endows it with the potential to interact with endometrial cells [95].

Studies in rodents suggest that implantation interactions between the embryo and the endometrium are restricted to only a short period of less than 24 h [107]. In women, the implantation window is considered to be longer, probably between days 20 and 24 of a standard menstrual cycle (or 7–10 days after ovulation) [32, 143]. Upon closure of the implantation window, the endometrium becomes refractory and hostile to unimplanted embryos.

In general, the luminal epithelium of the human uterus shows an apical glycocalyx that enables diffusion of small molecules but is suggested to protect against cell adhesion and bacterial infection in the upper tract [6, 8]. Among the anti-adhesive glycoproteins, large glycosylated mucins, such as MUC1 and MUC4, are considered important regulators of uterine receptivity [107]. Respective concepts suggest that during the pre-receptive phase, mucins mask potential adhesive ligands that are only demasked in the receptive window to allow bridging of the blastocyst to the endometrial epithelium. Studies in mice and rats, but also in vitro studies with hatched human blastocysts and endometrial epithelial cell monolayer cultures, indicate that anti-adhesive mucins disappear precisely or get at least modified at the time of expected implantation when the endometrium becomes receptive [47, 102, 126]. Interestingly, in vitro studies show that disappearance of MUC1 is confined to areas beneath and close to the attached embryo, while normal mucin expression persists in neighboring endometrial epithelial cells [9, 128]. Transition to the receptive phase of the endometrium is also reflected in ultrastructural changes, including gradual loss of epithelial cell polarity and formation of micro-protrusions on the apical surface called pinopodes or uterodomes [32, 43, 141].

From rodents, it is known that the period of highest endometrial receptivity coincides with the important process of blastocyst activation. Blastocyst activation is defined as the stage when the blastocyst acquires implantation competency in response to hydroxyestradiol-17β that is produced locally in the endometrium as a metabolite of estradiol [8, 107, 112, 147]. By local expression of adhesion molecules, the activated blastocyst undergoes apposition to the endometrial epithelium, a process suggested to have many features in common with leukocytes rolling along the vascular endothelium [58]. However, apposition is only a weak interaction of the trophectoderm with the uterine epithelium that is unstable to shear stress. Apposition is also reversible and thus allows repositioning of the blastocyst in the uterus [142, 168].

Initial interaction is suggested to be mediated by carbohydrate-binding glycoproteins, such as galectins and selectins, which are expressed on the trophectoderm, but not the inner cell mass of the hatched blastocyst [59, 107]. Observations in human hatched blastocysts and endometrial biopsies suggest that L-selectin and its binding ligands play a crucial role in this initial step [71]. Consistent with this finding, L-selectin ligands are expressed on pinopodes of human endometrial epithelium in the mid-luteal phase, on top of which blastocyst attachment is suggested to take place [14, 15, 138]. Once the initial interaction is established, the blastocyst—in particular the trophectoderm—may release soluble factors that are taken up by endometrial epithelial cells and potentially regulate their adhesive capacity via targeting gene and protein expression [41, 42].

This juxtacrine signaling shortly before and during the attachment reaction is thought to coordinate expression of cell adhesion molecules, such as heparin-binding EGF-like growth factor (HBEGF), trophinin and integrins, which contributes to a stable, shear stress-resistant adhesion of the blastocyst. HBEGF is expressed as a transmembrane form by the uterine epithelium and binds to heparan sulfate proteoglycan as well as to EGF receptor family members ErbB1 and ErbB4, all three of which are expressed on the trophectoderm of the hatched blastocyst [37, 146, 148, 155, 171]. ErbB4 tyrosine phosphorylation is reported to be required for trophectoderm differentiation into an invasive trophoblast phenotype, which is, however, inhibited in presence of the transmembrane protein trophinin and its associated cytoplasmic proteins bystin and tastin [5, 68]. Trophinin is expressed in both the trophectoderm as well as in the uterine epithelium [66, 67], and once homophilic binding of trophinin between trophectoderm and endometrial epithelial cells is established, bystin dissociates to enable ErbB4 autophosphorylation in the trophectoderm [175]. While homophilic trophinin binding triggers trophectoderm differentiation in the attached blastocyst, it is proposed to be at least partially involved in the process of trophectoderm contact-induced apoptosis of the endometrial epithelial cells [178]. In addition to trophinin, the Fas/Fas-L death system is proposed to locally induce apoptosis of endometrial epithelial cells to breach the uterine epithelial barrier [69, 83]. The loss of uterine epithelial cells adjacent to the blastocyst attachment site gives rise to exposure of extracellular matrix (ECM) components of the underlying basement membrane. Now trophoblast attachment to the ECM appears to be mediated predominantly by integrins that strengthen adhesion. Indeed, human pre-implantation embryos at the blastocyst stage show a number of integrin subunits, including α_3_, α_v_, β_1_, β_3_, and β_5_ [27]. With activation of the blastocyst, integrins may translocate to the apical surface of the trophectoderm, as has been suggested for α_5_β_1_ in mice [163]. However, in the rabbit model, the three isoforms α_1_β_1_, α_4_β_1_ and α_v_β_3_ have been suggested to be mainly involved in implantation, with α_v_β_3_ apparently playing a more predominant role than the others [88]. In human, the α_v_β_3_ dimer is present in both the uterine epithelium and the external surface of the expanded blastocyst, allowing bridging of the embryo to the uterine wall by ligands, such as fibronectin, laminin, perlecan, vitronectin, tenascin, and thrombospondin [106]. Another intensively studied bridging ligand hypothesized to play significant roles in mammalian implantation is osteopontin [93]. Osteopontin (encoded by secreted phosphoprotein 1, *SPP1*) is a secreted ECM protein that is up-regulated during the initial stages of pregnancy in the uteri of humans and many other mammalian species, including pigs, ruminants, rodents, and primates [57]. Beside its bridging function, osteopontin is suggested to induce downstream signaling pathways that regulate adhesion, cytoskeletal remodeling and migration of trophectoderm cells [105].

## Implantation period and initial formation of the syncytiotrophoblast

As outlined above, the trophectoderm cells of the blastocyst are engaged in apposition and adhesion of the blastocyst to the receptive luminal epithelium of the uterus. These processes lead to a close and stable contact between embryonic and maternal tissues, which results in the formation of the placenta. Only after proper apposition and adhesion of the blastocyst, the trophectoderm cells start their next differentiation steps during implantation.

**Fig. 2:**
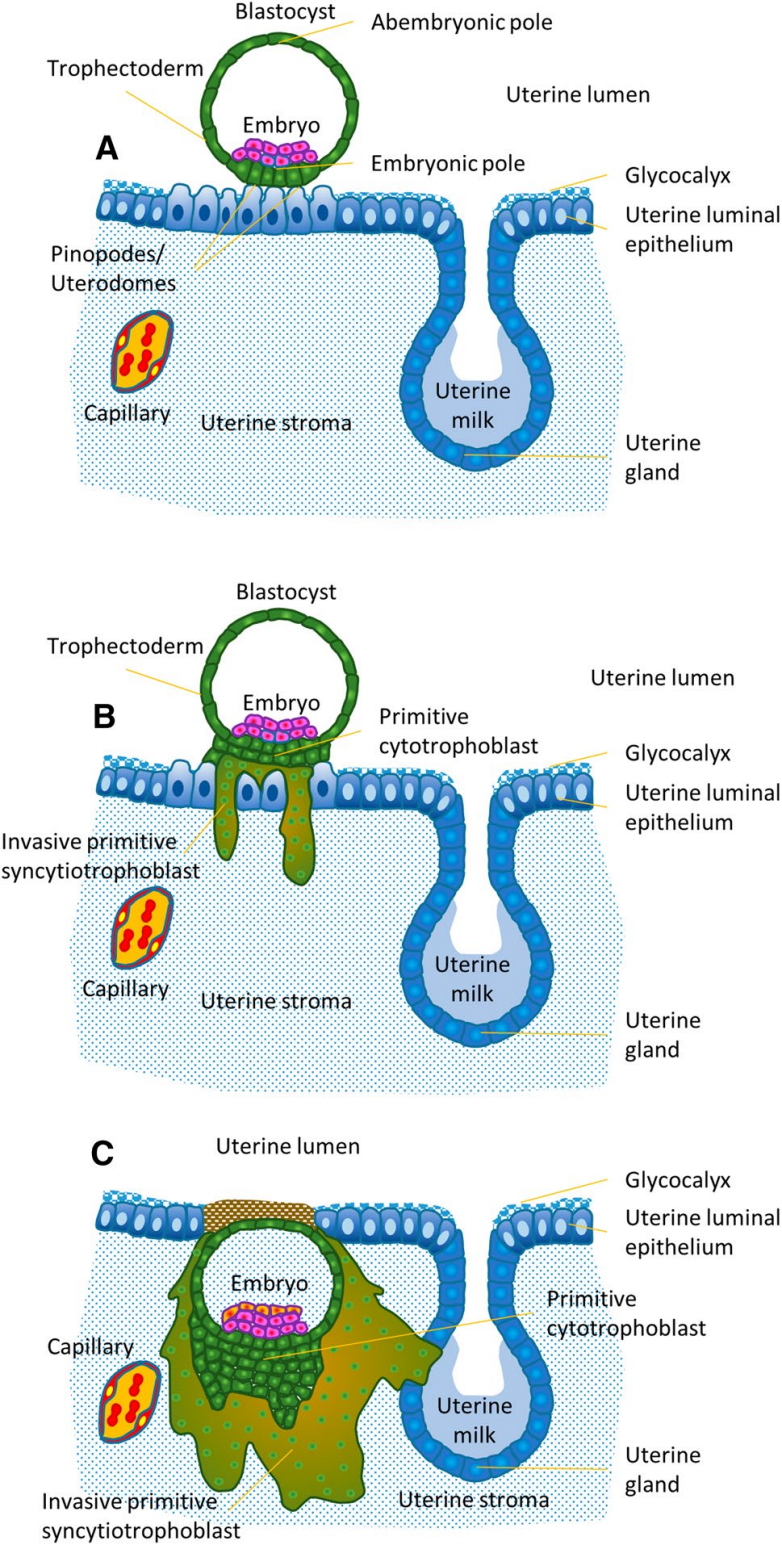
Implantation and initial syncytium formation. **A** The blastocyst is attached to the uterine epithelium. This epithelium is covered by a massive glycocalyx. At the site of blastocyst attachment, the pinopodes of the uterine epithelial cells allow direct interaction between these cells and the trophectoderm cells of the embryonic pole of the blastocyst, bypassing the glycocalyx. **B** At the embryonic pole of the blastocyst, the trophectoderm cells start to proliferate and form multiple cell layers of daughter cells, the primitive cytotrophoblasts. Some of the primitive cytotrophoblasts further differentiate and fuse with each other to generate the primitive syncytiotrophoblast, a multinucleated trophoblast structure. Only this primitive syncytiotrophoblast seems to be able to penetrate the uterine epithelium, allowing invasion into the uterine stroma. **C** By means of the invading primitive syncytiotrophoblast, the embryo has passed the uterine epithelium and is now fully embedded in the uterine stroma. The primitive syncytiotrophoblast fully surrounds the underlying mono-nucleated cytotrophoblasts as well as the embryo and thus is the only embryonic layer in contact with maternal tissues. Already at this stage of placental development, the primitive syncytiotrophoblast invades into a uterine gland to allow nutritive support of the embryo using glandular secretion products, called uterine milk

One of the key differentiation steps to enable invasion of the early embryo into uterine tissues is the formation of the primitive syncytiotrophoblast. Although formation of the primitive syncytiotrophoblast at this stage of human embryonic development is only anticipated using the approaches described below, it is more than tempting to speculate that this step is crucial for implantation in the human as well. Those trophectoderm cells at the embryonic pole that come into direct contact with uterine epithelial cells (Fig. 2A), start proliferation and differentiation. The daughter cells, now termed cytotrophoblasts, initiate cell–cell fusion and thus generate a cell with more than one nucleus. Further fusion events with this multinucleated cell result in the formation of the primitive syncytiotrophoblast, a multinucleated layer that has lost its proliferative capacity (Fig. 2B). It seems as if only this multinucleated layer is able to penetrate the uterine epithelium and allows embedding of the embryo into the uterine stroma (Fig. 2B, C). So far, it is not clear whether multiple primitive syncytia are generated at the very beginning that fuse to become one primitive syncytiotrophoblast, or whether there is the formation of only one primitive syncytiotrophoblast right from the beginning.

So far, the initial phases of trophectoderm penetration through the uterine epithelium have not been observed in the human in vivo. Thus, not only the underlying processes, but also the morphological changes of the trophectoderm cells at that specific time of embryo development have not been assessed so far. So far, processes and cellular structures of human implantation have been deduced using three different approaches:

1. Identifying structures using historical tissue slides that depict human implantation stages starting from day one after implantation [82, 144]. Allen Enders has generously given permission to view the images of his unique and extensive collection of placental material (https://www.trophoblast.cam.ac.uk/Resources/enders). The earliest human specimens in this collection are from day one after initiation of implantation and show embryos that have already crossed the uterine epithelium. Hence, although such images cannot tell us anything about the processes leading to breaching the uterine epithelium, they can display some interesting aspects of the embryo and especially the placenta at this stage of development.

In the collection of Allen Enders, there are three specimens from Carnegie stage 5A and thus should have a post-fertilization age of 7–8 days. The findings of the three cases (cases # 8020, # 8155 and # 8225) can be summarized as follows:

- The primitive syncytiotrophoblast is invasive and is always at the forefront of the embryo penetrating into uterine tissues. The primitive syncytiotrophoblast is also the extra-embryonic tissue taking over most of the contact area between embryonic and maternal tissues at this stage of pregnancy.
- Presence of multiple primitive syncytiotrophoblast masses invading into the uterine stroma. This may indicate that there is more than one initial fusion event between two primitive cytotrophoblasts resulting in the formation of a syncytium. However, since these are single sections of a three-dimensional structure, the multiple syncytial masses may still be parts of a single primitive syncytiotrophoblast when looking at the complete 3D structure. Later in gestation, when the syncytiotrophoblast has further differentiated into the coverage of the villous tree, there is only one single multinucleated layer that is constantly fed by fusion of underlying villous cytotrophoblasts. Only the very early initial fusion events occur between two primitive cytotrophoblasts. Later in pregnancy, syncytial fusion of two villous cytotrophoblasts no longer occurs, as there is no second syncytial layer in the human placenta different to the rat and mouse placenta that show two layers of syncytiotrophoblast.
- While invading into the uterine stroma, the primitive syncytiotrophoblast comes into close contact with small capillaries as well as larger uterine glands. One image of case # 8020 shows that the primitive syncytiotrophoblast has already invaded into a uterine gland, while there is no indication of invasion into blood vessels at this stage of development.

Another source for such historical tissue slides depicting human implantation stages is the “Virtual Human Embryo” collection of Raymond Gasser (https://www.ehd.org/virtual-human-embryo).

(2) Deploying comparative placentology to identify respective structures in other animals, especially non-human primates [56, 169]. Again, the images of Allen Enders’ collection of placental material give a deep insight into the implantation process in monkeys, such as the baboon or the macaque (https://www.trophoblast.cam.ac.uk/Resources/enders).

For the macaque, images are available that depict the first day of implantation. These images clearly show that it is the primitive syncytiotrophoblast that penetrates through the uterine epithelium, while the primitive cytotrophoblasts remain in second row behind the syncytial masses. Interestingly, in the macaque the primitive syncytiotrophoblast extends into the layer of the uterine epithelial cells and then seems to rest on the basement membrane of this epithelium pushing the epithelial cells toward the margin of this area or even completely surrounding such cells. Also, at the second day of implantation, the primitive syncytiotrophoblast remains on the epithelial basement membrane and may use this layer to move into adjacent uterine glands, eliminating the glandular epithelium on its way into the gland. Only in the early lacunar stage of placental development, the primitive syncytiotrophoblast invades into the uterine stroma and opens capillaries [52, 53].

This description already reveals obvious differences even between human and primates such as the macaque implantation. Hence, a direct interpolation from the animal to the human situation should be done with great caution.

(3) Performing in vitro studies that use blastocysts or cell lines (human or other animals) and endometrial epithelial cells or cell lines (again human or other animals) [13, 17, 98, 158].

Quite a number of studies has shown direct interactions between a human blastocyst and luminal epithelial cells of the uterus in vitro [1, 13, 97, 121, 128, 158]. However, since the blastocysts were cultured on a cell monolayer, the studies focused primarily on adhesion of the blastocyst to the epithelial cells rather than identifying penetration of trophoblast structures through the epithelial layer. The study of Bentin-Ley et al. [13] performed ultrastructural analysis of blastocysts that attached to epithelial cells. Using this technique, the authors identified desmosomes between trophectoderm and epithelial cells as well as the formation of first syncytia. The recent study of Ruane et al. [159] used human blastocysts that were cultured on Ishikawa cells, a human endometrial adenocarcinoma cell line. These authors visualized multiple trophectoderm-derived syncytia penetrating through the Ishikawa cell layer and identified the epithelial–trophoblast interactome during this crucial step of embryo development. So far, the knowledge on the blastocyst breaching the uterine epithelium in the human is still highly fragmented.

## Villous formation and the villous trophoblast population

- Villous formation

The placental villous trees represent the main functional units of the placenta [73, 122], which is why their adequate formation is crucial for the development of a healthy fetus [190]. The early villous formation phase starts within 12 to 18 days post conception (p.c.) [29, 108] and lasts until day 28 p.c., followed by the villous phase which lasts until the end of pregnancy [12, 84]. During early formation of placental villi, i.e., during the lacunar phase of placental development, the differentiation of the villous trophoblast results in two layers consisting of the outer villous syncytiotrophoblast and the underlying, proliferating villous cytotrophoblasts. Around day 14 p.c., cells of the extra-embryonic mesoderm originating from the embryo form an additional cell layer underneath the primitive cytotrophoblast, which will later develop into the inner stromal core of placental villi [29, 85]. Concomitantly, the cytotrophoblasts start to invade into the syncytial trabeculae forming finger-like outgrows filled by mononuclear cytotrophoblasts, surrounded by the multinuclear syncytiotrophoblast layer. This formation is also known as trophoblastic sprouting and the structure represents the primary villi which consist of villous trophoblast only [85, 108]. Subsequently, the extra-embryonic mesoderm follows the cytotrophoblasts and invades the primary villi. Mesenchymal cells supersede the cytotrophoblast core, transforming them into secondary villi [85]. These secondary villi consist of two trophoblast layers (outer syncytiotrophoblast, inner cytotrophoblast) filled with a mesenchymal core [84, 108, 110]. Vasculogenesis, by means of de novo formation of blood vessels within the mesenchyme of the secondary villi begins at 18–20 days p.c., which remodels them into tertiary villi [46, 84, 108, 110, 131]. This early phase of vasculogenesis occurs independent from the development of vessels and blood cells in the embryo. Mostly, all of the tertiary villi will be vascularized by the end of the first trimester [12, 84, 108]. The formation of new villi and thus the continuous growth of the villous trees last until the end of pregnancy following the uniform process as described above (1. trophoblast sprouting—primary villi, 2. mesenchymal invasion—secondary villi, 3. local angiogenesis—tertiary villi) [29, 30, 84]. Interestingly, toward the end of the first trimester (around weeks 8–11), a premature flow of maternal blood into the abembryonic part of the placenta results in regression of placental villi at this site, maybe because of an untimely increase of oxygenation [91].

(b) Villous trophoblast population

As stated above, the placenta undergoes a considerable transformation within the first trimester of pregnancy. It represents an exceedingly cellular heterogeneous and dynamic organ leading to an intriguing field of research [122]. Single-cell RNA sequencing (scRNA-seq) analysis represents a promising tool to identify new cell subtypes of the early human placenta and may enlarge our understating of early human placental development and functions [122]. Recent studies provide a blueprint of the dynamic changes within placental cells and their composition, thus inaugurating evidences for different roles of newly identified subtypes of villous trophoblasts in early gestation [122, 176]. Suryawanshi et al. showed transcriptome definitions of 9 cell populations from villous cell types, including villous cytotrophoblast, syncytiotrophoblast, extravillous trophoblast, three fibroblast cell clusters, vascular endothelial cells, erythroblasts, and Hofbauer cells [176]. In the first trimester, the proportion of trophoblasts is about 41%, while villous cytotrophoblast represents the most abundant trophoblast cell type throughout gestation followed by syncytiotrophoblast and extravillous trophoblasts (based on the number of nuclei) [176]. Concomitantly with another study, analysis showed a proliferating subpopulation of villous cytotrophoblasts [122, 176]. The authors attributed this subpopulation to reflect the differentiation of villous cytotrophoblast to extravillous trophoblasts [176], while the other study assigned them to represent the reservoir that refills the cytotrophoblast pool [122]. Moreover, two additional non-proliferative villous cytotrophoblast subtypes where found. One not expressing syncytin-2 (encoded by *ERVFRD-1*) while the other subtype showed to be syncytin-2 positive, serving as a progenitor cell of the syncytiotrophoblast [122].

(iii) Function of villous trophoblast subpopulations

During early gestation, the general histological structure of the placental villi results in the placental barrier consisting of the villous syncytiotrophoblast, villous cytotrophoblasts, a trophoblastic basal membrane, mesenchymal stroma and placental blood vessels without a basement membrane. Later in gestation, the placental blood vessels are also covered by a basement membrane [12, 84]. Apart from the barrier function between maternal and fetal blood circulation, the placental villi, in particular the outer syncytiotrophoblast, have a significant role in the fetal–maternal gas and nutrient exchange as well as endocrine and metabolic activity [23, 73]. Microvilli at the apical side of the syncytiotrophoblast enlarge the surface area to increase the exchange ability [12, 84]. The constant fusion of villous cytotrophoblasts with the syncytiotrophoblast maintains the highly active metabolic status of the syncytiotrophoblast through the steady transfer of new nuclei, mRNA transcripts and organelles [108].

Furthermore, the syncytiotrophoblast produces a variety of hormones from first trimester of pregnancy onwards, affecting the cross talk at the maternal–fetal interface [63, 108, 110]. Human chorionic gonadotropin (hCG) is mainly synthesized in the syncytiotrophoblast. The hCG levels exponentially rise during the first 7 weeks of pregnancy, which is important for the regulation of the hemochorial placentation and placental and fetal growth [38, 63]. Moreover, it has been shown, that hCG provokes the differentiation of trophoblasts and the fusion of cytotrophoblasts with the syncytiotrophoblast [63, 166]. Additionally, a study showed a positive feedback loop between hCG triggered placental development and hCG production [36, 63]. Notably, maternal platelets adhere at the surface of the syncytiotrophoblast of first trimester placental villi [19, 75] and can also be found in interstices of trophoblast cell columns of anchoring villi [74, 75, 131]. In this manner, platelets and platelet-derived factors may well affect villous trophoblast physiology, which for example has been shown by the impairment of hCG synthesis and secretion [63].

Beside hormones, the growing syncytiotrophoblast releases waste products and particles into the maternal body, modulating feto-maternal signaling [24, 160]. An adequate interaction between placenta-released particles and hormones into the maternal circulation and the response of the mother to these factors is a key mechanism for uncomplicated pregnancies [84]. With progressing pregnancy, apoptotic structures, known as syncytial knots, are released from the outer surface of the placenta carrying multiple late apoptotic nuclei [85] and then get stuck in the capillary bed of the maternal lungs [12, 85, 87]. The frequency of releasing syncytial knots from the surface of the villous syncytiotrophoblast increases with gestational age [25, 62, 123]. Importantly, this process is the last step in the villous trophoblast turnover and typifies a regular turnover as seen in other stratified epithelia rather than triggering a maternal inflammatory response [86]. It should be noted that according to literature, the term syncytial nuclear aggregates (SNAs) is often used as a collective term of a heterogeneous group of syncytiotrophoblast-derived aggregates of syncytial nuclei including syncytial sprouts and apoptotic syncytial knots. The definition of the different types by light microscopy is challenging, resulting in variations of the nomenclature in literature. Moreover, the terms syncytial knots and syncytial sprouts are often used synonymously. However, there is evidence that the individual syncytiotrophoblastic structures differ significantly from one another [25]. Syncytial sprouts differ in at least three key aspects from syncytial knots. First, euchromatic nuclei with no evidence of degeneration are found within the sprouts. Second, sprouts are linked to cytotrophoblast proliferation and formation of new villi, which is why they are mostly found in earlier gestational age. Third, syncytial sprouts are mostly pedunculated [25]. Generally, more sprouts are found in first trimester placentas whereas syncytial knots are mostly found toward term.

Concomitantly with syncytial knots, extracellular vesicles (EVs) are also released from the syncytiotrophoblast from the first trimester of pregnancy [114] with increasing amounts until term [109, 115]. They are carrying transcriptionally active and immunomodulatory cargo from the placenta to the maternal body [114, 145, 156, 181], where the cardiovascular system seems to be one of the main targets [2, 179]. It seems as if such EVs also have a positive effect on the invasiveness of extravillous trophoblasts [177]. Of note, signaling between mother and fetus occurs in a bidirectional manner [136], which is why also maternally derived EVs affect implantation as well as physiological functions of trophoblasts and thus placental development [72, 139, 198]. Interestingly, in vitro, internalized endometrial EVs by HTR8 trophoblast cells promote their adhesive capacity [72]. In vivo, this could increase the capability of trophoblasts to attach to the endometrium [72]. Moreover, EVs seem to be involved in adaption of the maternal physiology to ongoing pregnancy. Although placenta-derived EVs represent a challenging task for the mother, they also are suggested to prime the maternal immune and cardiovascular system to pregnancy [40, 137, 182]. To this end, the highly dynamic and extremely regulated interaction between first trimester placenta and maternal cells determines the establishment of a healthy pregnancy [125, 176, 180].

## Extravillous trophoblast subpopulations and trophoblast invasion

As outlined above, primary villi start to emerge from day 13 p.c. and consequently result in the formation of primitive villous trees. Some stems of those villous trees remain attached to the maternal side, where they are called anchoring villi. This villous type does not only facilitate mechanical stability but is also the source for the extravillous trophoblast populations [54, 55]. The connection between an anchoring villus and the uterine decidua basalis is characterized by a cell column of mononuclear extravillous trophoblasts (EVTs). At the very tip of anchoring villi, there is no covering syncytiotrophoblast. Hence, proliferation of cytotrophoblasts results in the formation of multiple layers of daughter cells, called trophoblast cell column. During the first trimester of pregnancy, some of the cell columns still seem to have a surrounding syncytiotrophoblast layer [55]. The cell columns show a differentiation gradient of trophoblasts from the proximal part attached to the basement membrane of the anchoring villus down to the distal part within the uterine stroma. The most proximal EVTs, attached to the basement membrane of the anchoring villus, are of similar character to villous cytotrophoblasts and express the typical epithelial integrin types α3β1 and α6β4 [7, 44]. The EVTs of this first layer are of the proliferating phenotype [103] and their daughter cells are arranged in up to 6 layers in first trimester cell columns, forming a compact zone with little intercellular space that largely lacks ECM components [64]. EVTs of the proliferating type express EGFR and ERBB4, but are devoid of ERBB2 and ERBB3 [61]. For a long period, it has been believed that by exiting the high proliferation state EVTs leave the cell cycle [100], although immunoreactivity for the proliferation marker Mib-1 is still observable [103], and α6β4 integrins are still expressed [44]. However, recent observations suggest that during the differentiation of a large number of EVTs, a tetraploidization of their genome occurs, which is independent of mitotic cycles [188]. This process of endoreduplication is known from different organisms and plays an important role in development [120] (e.g., rodent placental giant trophoblast cells [10]). The endocycle-dependent genome amplification remains throughout EVT differentiation and is finally lost by switching to the invasive phenotype when EVTs exit the distal cell column and enter a senescent state [188]. In accordance with these findings, Liu et al. showed with scRNA-seq that the EVTs from the proximal portion of the cell column have an increased expression of ribonucleotide reductase regulatory subunit M2 (RRM2), which plays a role in DNA replication [122]. Both, the proliferating and post-proliferating, EVT populations show keratin 7 immunoreactivity [103], as do all trophoblast subtypes. Beneath the compact zone, the EVTs become larger and polygonally shaped with irregular microvilli-like processes and cytoplasmic glycogen particles [118]. There is more intercellular space between the cells than in the compact zone enabling a possible route for maternal plasma components, including particles, such as platelets and multi-vesicular cargos entering the early intervillous space [74]. In this transition zone, the EVTs are embedded in a self-secreted matrix of laminins, collagen IV, vitronectin, heparan sulfate, cellular fibronectins including oncofetal isoforms and fibrillin [64, 99, 103]. This matrix is termed “matrix-type fibrinoid” [99]. Here, the cells are not of the ‘true’ invasive character as they still express α6β4 integrins but also the α5β1 integrins, the ‘interstitial integrin’ [44, 45]. Based on scRNA seq data, at least two different subtypes of EVTs in the distal portion of the cell column are found, plasminogen activator inhibitor-1 (PAI-1, encoded by *SERPINE1*) positive and negative ones [122]. Acquisition of the invasive extravillous trophoblast phenotype is associated with an integrin switch from α6β4 (proliferative phenotype) to α1β1 (invasive phenotype) [45]. Recently, a member of fermitin family of adapter proteins (FERMT) has been suggested to regulate integrin activation and the subsequent integrin-mediated signaling in EVTs [101]. Beside the integrin switch, EVTs in the distal cell column start expressing markers, such as HLA-G, ERBB2 and NOTCH2 [61, 76]. The intercellular space between the EVTs increases toward the distal end of the cell column. The cell size and the amount of glycogen of the non-invading EVTs increases and the cells become morphologically identical with those found in the basal plate in a term placenta [54, 118]. At the same time, the invasive phenotype of EVTs shows a spindle-shaped morphology similar to invasive tumor cells [103].

When extravillous trophoblasts leave the trophoblast cell columns, they switch into an invasive phenotype, and a subpopulation of which shows cellular senescence (growth arrest), a high senescence-associated beta-galactosidase activity, and a tetraploidy. Moreover, this phenotype shows metabolic alterations, including pronounced glycogen storages and increased fatty acid synthesis. The tetraploidy and metabolic changes during the differentiation process of EVTs result in increased nuclear and cellular volume [188]. Beside the large polygonal EVT population, a small spindle-shaped EVT population is found along the whole invasive pathway which show integrins α5β1, αvβ3/5 [103].

Importantly, extravillous column outgrowth is enhanced by a low oxygen environment, which promotes the expression of genes that align with EVT lineage commitment. Differential gene expression analysis of column trophoblast samples cultured under both 1% and 5% oxygen, revealed top hits including genes associated with hypoxia (EGLN3, RORA), cell–matrix interaction and/or re-structuring (LOX, JAM2, EGLN3, PLAUR) and gene transcription regulation (MXI1, TSC22D3, RORA) [183]. While these data were generated by a Matrigel-embedded placental explant model, recent progress in the establishment of trophoblast organoids that can differentiate into the two main trophoblast cell lineages [77, 184] will allow for further deep mechanistic examination of involved pathways.

Beside senescence and tetraploidy, additional molecular changes take place to enable EVTs to migrate into the decidua. Among these, secretion of metalloproteinases and genes associated with epithelial-to-mesenchymal transition (EMT) are up-regulated, whereas junction proteins are down-regulated. Moreover, EVT differentiation is suggested to be associated with the induction of key transcription factors of EMT such as SNAIL controlled by upstream signaling cascades, including FGF, TGF-β and Wnt [50]. However, EMT in EVTs is not fully established since the hallmark marker of epithelial trophoblasts, keratin 7, is not down-regulated and contrary the classical mesenchymal marker vimentin, is not induced during EVT formation [50].

**Fig. 3:**
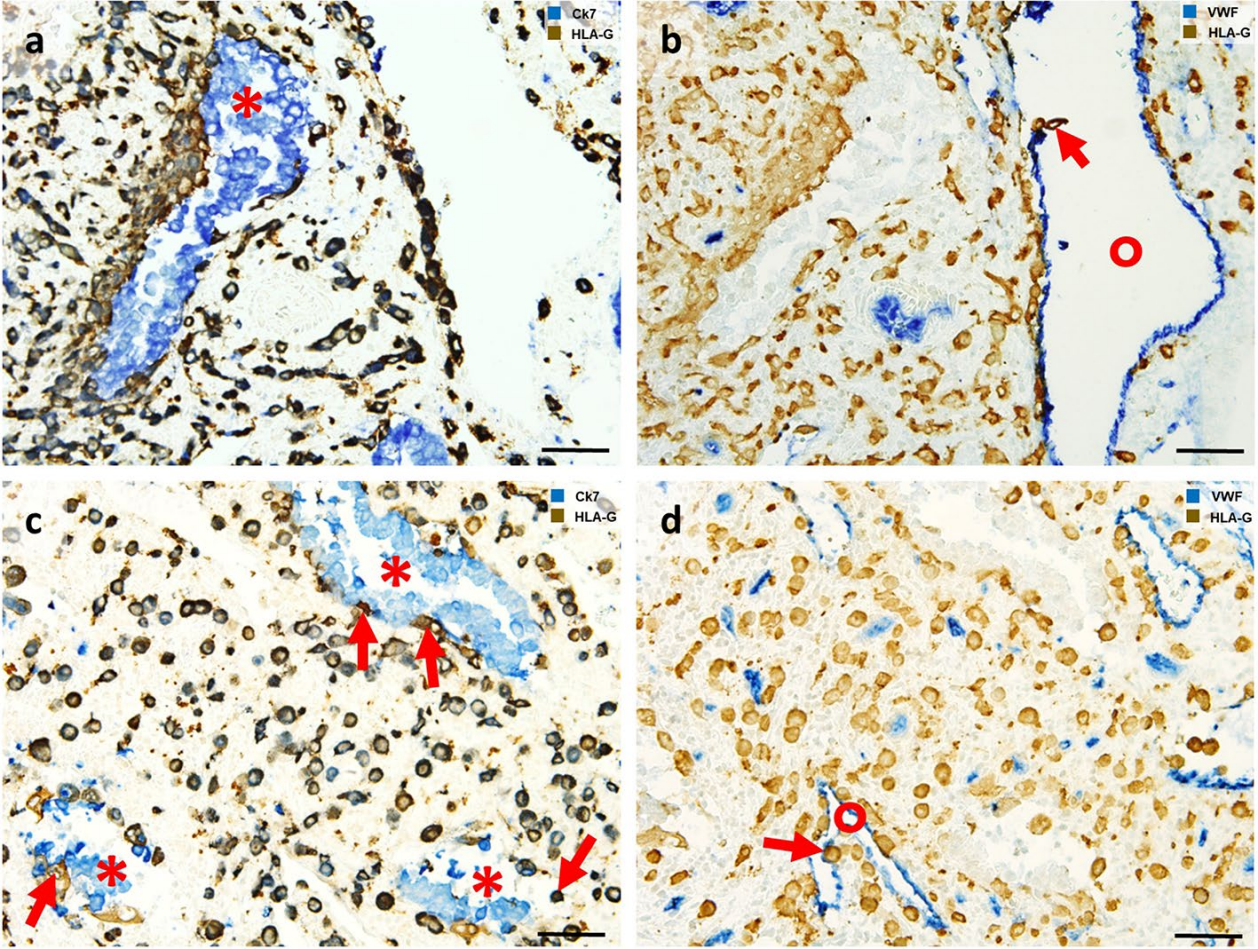
*Extravillous trophoblast invasion: The interstitial, endoglandular and endovascular trophoblast subtypes*. Rows (**a**, **b** and **c**, **d**): Serial sections from first trimester decidua basalis (gestational age 7 weeks). Columns (**a**, **c**, **b**, **d**): Immuno double staining for HLA-G (dark brown) and keratin 7 (blue) (**a**, **c**) or HLA-G (brown) and von Willebrand factor (blue) (**b**, **d**). EVTs (brown) appear spindle-shaped or polygonal within the decidual stroma (**a**–**d**). Glands (red asterisks) are invaded by endoglandular EVTs (arrows) from the interstitial side, glandular epithelium (blue in **a**, **c**) looks disintegrated in the invaded regions (**a**, **c**). EVTs (arrows) are also present within all types of uterine vessels (circles, endothelium stained in blue) (**b**, **d**). A clear differentiation of endovascular subtypes is not possible in such small vessels. Scale bar represents 50 µm, no nuclear counterstain

Invasion into the maternal decidua meets two important requirements of successful human placentation. First, EVT invasion guarantees anchorage of the placenta within the uterus (via interstitial trophoblasts), and second, EVTs erode uterine vessels and glands to allow fetal supply with nutrients and oxygen (endoarterial, endovenous, endolymphatic and endoglandular trophoblasts) (Fig. 3). Depending on location and function, there are different types of EVTs described along the invasive pathway.

- The interstitial EVT population

Dispersed in the decidual stroma of the placental bed, mononuclear interstitial EVTs appear in large numbers usually as small spindle-shaped cells (invasive) and large polygonal cells (non-invasive). Their common features are the expression of keratin 7 and major histocompatibility complex, class I, G (HLA-G) on their surface, beside general markers for invasion, cell–cell interaction and extracellular matrix degradation (such as integrins, and matrix metalloproteases) [12]. Recently, placenta-specific protein 8 (PLAC8) was presented as a specific marker for interstitial EVT [33]. They invade as far as the inner third of the myometrium. At the border between decidua and myometrium, a large number of these cells transform into multinucleated giant trophoblasts (also referred to as ‘giant cells’) [103]. Multinucleated giant trophoblasts are characterized by their large size and display clusters of nuclei surrounded by ample cytoplasm that only focally shows keratin 7 immunoreactivity. It is suggested, that they are formed within a terminal differential step during a progressive reduction of the invasive population and so far, no active function is known. Interestingly, even desmosomal contacts between giant cells and mononuclear EVTs have been described and the giant cells resemble characteristics of the villous syncytiotrophoblast in many ways (microvilli, occasionally secretory droplets) [94] and may also be generated by fusion of mononucleated trophoblasts. No specific transcriptome signature of this cell phenotype has been found by scRNA seq of placental bed tissue [189].

(b) The endovascular EVT populations

### Endoarterial trophoblast

Beside other cell populations, endoarterial EVTs contribute to the complex process of uterine spiral artery remodeling [79]. This process is characterized by vascular smooth muscle cell separation and loss, vessel dilatation and invasion of EVTs. Uterine spiral artery remodeling is an essential process for a successful pregnancy. It takes place in more or less parallel settings: trophoblast-independent spiral artery remodeling is triggered by progesterone dependent decidualization and goes along with partial loss of arterial muscular lining together with endothelial vacuolation and swelling. Decidual natural killer (dNK) cells and macrophages are considered to play a major regulatory role in this process [119, 150, 152, 170]. Meanwhile, EVTs reach the spiral arteries from the interstitial side (sometimes also called perivascular trophoblasts or—if present in the vessel wall—intramural trophoblasts) and embed themselves in fibrinoid material within the arterial walls. Endoarterial trophoblasts may partly replace the endothelium and further disrupt the arterial wall and finally open the spiral arteries as large and stiff tubes toward the intervillous space with all its physiological consequences in terms of blood flow toward the placenta [26, 162, 167]. It is plausible that some of the endoarterial trophoblasts crawl on top of the endothelium and may invade into the wall at a distant site again. The more trophoblast cell columns develop during placentation, the more EVTs can reach the spiral arteries from various sites.

Various players, such as proteins, cytokines, transcription factors and microRNAs, have influence on the process of endovascular trophoblast invasion and respective mechanisms are continuously presented and published. So far, only data for the regulation of endoarterial trophoblast invasion are available. For example, achaete-scute family BHLH transcription factor 2 (ASCL2) was confirmed as a critical and conserved regulator of the invasive trophoblast cell lineage and *ASCL2* expression was especially prominent in the endovascular EVT population [186]. Besides, it was demonstrated that also microRNAs are capable of influencing endoarterial trophoblast differentiation and spiral artery remodeling [20, 80]. In these studies, the authors usually do not stratify/differentiate between endoarterial, endovenous and endolymphatic trophoblasts.

Within the spiral arteries endoarterial EVTs aggregate to trophoblast plugs. These aggregates transiently inhibit the blood flow to the placenta in early pregnancy and thus have an important impact on the utero-placental hemodynamics. They persist in the decidual and myometrial parts of the spiral arteries beyond the first trimester [4, 151]. This challenges the current opinion, that spiral artery remodeling and trophoblast plugging are exclusive events of the first trimester [4]. Despite building a physiological barrier to restrict flow of maternal blood cells into the placental intervillous space, trophoblast plugs are not completely leak-tight, there are channels within the plugs that enable an early microvascular filling of the intervillous space with blood plasma [4, 90, 157]. However, along with disintegration of plugs and establishment of the utero-placental blood flow at about 11 weeks of gestation, a rapid increase of oxygen in the intervillous space occurs [92].

It is still controversially discussed whether EVTs really fully replace the arterial endothelium and take over endothelial characteristics or not. Although HTR8 trophoblast cells in vitro integrate into already existing endothelial cellular networks via integrins α1β1 and α6β4 [195]*,* it was recently demonstrated, that plugged vessels are still covered by more than two thirds with endothelial cells and that the so-called endothelial mimicry by EVTs does not occur [21]. However, recent in vitro model systems suggest that EVTs can induce endothelial cell death, or at least that endothelial cells are sensitive to apoptotic stimuli like TNF-α [34]. Other potential players during uterine spiral artery remodeling are immune cells (macrophages and dNK cells), especially dNK cells are present in high numbers within the decidua, and are suggested to be responsible for the disruption of endothelial structures [28, 65, 170]. A very novel organ-on-a-chip model supports a general promoting role of dNK cells on EVT invasion [149].

### Endovenous and endolymphatic trophoblast

Formerly strictly allocated to the uterine spiral arteries, it is now clear that endovascular trophoblasts locate to all branches of the vascular system. Hence, they were described to be located in maternal veins (endovenous trophoblast) as well as lymphatic vessels (endolymphatic trophoblast) [81, 134, 193]. Uterine veins are even invaded prior to spiral arteries and connected to the intervillous space of the placenta to ensure fluid drainage from the placenta back into the maternal system [81, 134, 193]. Lymphatic vessels are invaded by endolymphatic trophoblasts resulting in the presence of EVTs in local lymph nodes [81]. Therefore, further stratification of endovascular trophoblast into endoarterial, endovenous and endolymphatic trophoblast is helpful in regard to differentially expressed cell-specific markers. As an example, CD56 is expressed on endoarterial trophoblasts within trophoblast plugs (= endoarterial trophoblast), but not on interstitial trophoblasts. Although this is just an estimation based on extensive microscopic experience, we suggest that endovenous and endolymphatic trophoblasts are phenotypically more closely related to interstitial and endoglandular trophoblasts than to endoarterial trophoblast [96, 135, 187].

The function of trophoblast invasion into veins and lymph vessels has been questioned in the very beginning when this was identified. Today, it has become obvious that invasion into uterine veins is essential for the flow of maternal blood through the placenta. All the fluids that enter the placenta from the maternal side (plasma and glandular secretion products in the first trimester, blood in the second and third trimesters) need to be drained back into the maternal system. This is achieved by connecting the uterine veins to the intervillous space of the placenta by means of endovenous trophoblast invasion [130]. The function of endolymphatic trophoblast invasion into uterine lymph vessels, however, is less understood. It has been speculated that invasion of lymph vessels supports drainage of maternal fluids from the uterus back into the maternal system and that endolymphatic EVTs in lymph vessels may regulate immune cell influx and efflux [193].

(iii) The endoglandular EVT population

The frequent connection between invading EVTs and uterine glands has been demonstrated in the last decade [130, 132, 133, 135]. During early pregnancy, EVTs invade toward the numerous uterine glands within the decidua, locate themselves within the glandular epithelium, reach the lumen of the glands and thereby connect the glands with the intervillous space of the placenta. Hence, this type of EVT invades the maternal uterine glands and thus enables flow of their glandular secretion products into the intervillous space of the placenta. Other than hitherto assumed, the uterine glands within the endometrium are no singular tubes, instead they have branches and connections and even form a plexus network within the stratum basalis and expand along the myometrium [196]. Although demonstrated so far only in non-pregnant endometrium, it is unlikely that this plexus network vanishes in the decidua with the beginning of pregnancy rather than persisting as a glandular network throughout (at least) early pregnancy. Thus, endoglandular trophoblast invasion serves to connect the placenta, precisely the early intervillous space, with the whole glandular plexus.

In regions of strong trophoblast invasion, the glandular epithelium appears often disintegrated, the glands partially lose their epithelial lining, likely due to secretion of paracrine factors secreted by interstitial and endoglandular trophoblasts. Pro-invasive proteins, such as matrix metalloproteinase (MMP)1, MMP9 and integrin β1, are expressed by endoglandular trophoblasts [129, 133, 192]; further factors still need to be investigated. Within the first trimester, even more cross sections of uterine glands than of vessels are invaded by invasive trophoblasts [133], which is well justified with the need of histiotrophic nutrition of the embryo before hemotrophic supply of the fetus. Factors secreted by the uterine glands (such as EGF and other so-called uterine milk components) are likely to stimulate and trigger trophoblast development and invasion. Details and evidence of the histiotrophic nutrition in the human development were recently reviewed by Burton et al. [22].

So far, the recent scRNA-seq approaches did not stratify between the above described phenotypes of invasive EVT. Hence, so far there is no information whether these subtypes display differences in their expression signatures [176, 189]. In most of the hitherto performed studies, it was not assessed whether markers for EVTs are differentially expressed on different EVT subtypes. As mentioned above, PLAC8 was presented as a novel marker for interstitial EVT but not for endoarterial EVT. However, it was not tested, whether its expression differs on endoglandular, endovenous or endolymphatic EVTs [33]. Additional research in this direction is definitely needed.

## Conclusion and outlook

Advancements in single-cell transcriptomics and multi-omics technologies enable profiling of thousands of human trophoblasts obtained either from first trimester placenta specimens, 3D organoids or bone morphogenetic protein 4 (BMP4)-exposed pluripotent stem cells. These novel approaches will contribute to more precisely characterize new sub-clusters of previously poorly described trophoblast subpopulations [11, 104, 153, 185, 194]. Integration of spatial transcriptomics and advanced imaging techniques will further enhance the characterization of the spatiotemporal distribution of these trophoblast sub-clusters during human placental development and will shed light on altered compositions of these clusters in placenta-associated pregnancy pathologies.

## Data Availability

Not applicable.
